# The effect of smoking on spontaneous passage of distal ureteral stones

**DOI:** 10.1186/1471-2490-14-27

**Published:** 2014-03-22

**Authors:** Adem Fazlioglu, Yilmaz Salman, Zafer Tandogdu, Fatih Osman Kurtulus, Serap Bas, Mete Cek

**Affiliations:** 1 Department of Urology, Taksim Teaching Hospital, Istanbul, Turkey; 2 Department of Radiology, Gaziosmanpasa Hospital, Istanbul, Turkey; 3 Department of Urology, Trakya Medical School, Edirne, Turkey

**Keywords:** Ureteral stone, Smoking habits, Nicotine, Distal stones, Spontaneous passage

## Abstract

**Background:**

Animal studies have shown that nicotine affects the peristalsis of the ureter. The aim of the study is to analyze the effect of smoking on spontaneous passage of distal ureteral stones.

**Methods:**

88 patients in whom distal ureteral stone below 10 mm diameter diagnosed with helical computerized tomography enhanced images were reviewed. Patients were grouped as either smokers (n:33) or non smokers (n:50). Follow-up for spontaneous passage of stones was limited with 4 weeks. Patients did not receive any additional medical treatment other than non-steroid anti inflamatory drugs only during painful renal colic episodes.

Two groups were compared with the chi-square test in terms of passing the stone or not. Stone passage was confirmed with either the patient collecting the stone during urination or by helical CT.

**Results:**

Smoking habits was present in 30(34%) patients and the frequency in both groups were similar (smokers: 23(76%) vs non-smokers: 46(79%)). Spontaneous passage of the stone was observed in 69(78%) patients. The two groups were comparable in terms of patien age, male to female ratio and stone size. Stone passage decreased as stone diameter increased. Total stone passage rates were similar in both groups (smokers: 76% vs. non-smokers: 79%) (p > 0.05). Passage of stones > 4 mm was observed in 46% and 67% of smokers and non-smokers respectively. However passage of stones with a diameter ≤ 4 mm were similar in both groups (smokers: 100% vs non-smokers: 92%) (p > 0.05).

**Conclusion:**

Smoking has neither a favorable nor un-favorable effect on spontaneous passage of distal ureteral stones. However, spontaneous passage rates in patients with a stone diameter > 4 mm was lower in smokers. These results should be further confirmed with studies including larger numbers of patients.

## Background

The prevalence of urolithiasis within the urinary tract is 2-3%. According to the current literature we know that stone localization and size are the most important factors associated with spontaneous stone passage. According to the European Association of Urology urolithiasis guidelines the rate of spontaneous passage for stones < =5 mm was 68% independent of location within the ureter [1].

Most of the time patients develop a colic style pain during the spontaneous passage of a urinary stone. In addition to the colic type pain, spontaneous passage of stones have also been associated with deterioration in renal function and increased susceptibility to urinary tract infections in some patients. During the management of ureteral stone the aforementioned possible outcomes should be taken into consideration. Waiting for the spontaneous passage of the stone with or without additional medical treatment is an option for a significant number of patients. In such cases the time frame suggested for the spontaneous passage is 4 weeks [1].

Many studies in the past have exploited various medical treatment options that could assist the spontaneous passage of distal ureteral stones. Alfa adrenergic receptor blockers, prostaglandin inhibitors, steroid treatment (e.g. Deflazacort 30 mg daily, methylprednisolone) and calcium channel blockers are some examples of the research undertaken [2,3]. Studies have shown that alfa-1 adrenergic receptor blockers inhibit the peristaltic activity and basal tonus of the ureter and increase the spontaneous passage rates significantly [4].

Nicotine, one of the major components of cigarettes, activates the sympathetic nervous system by acting on the nicotinic acetylcholine (ach) receptors. Boyarsky et al. hypothesized that nicotine may affect the ureteral peristalsis by the mentioned induction of the ach receptors. They tried to answer this question by exposing dogs to nicotine and have shown that inhaled, intravenous and topical nicotine all increase the peristalsis of dog’s ureter [5].

As mentioned before nicotine has a possible effect on ureteral peristalsis. We hypothised that absorbed nicotine in cigarette smokers would influence peristalsis of the ureter and affect the spontaneous passage of ureteral stones. In this study the effect of smoking on spontaneous passage of distal ureteral stones smaller than 10 mm has been evaluated.

## Patients and method

After ethical board approval from Gaziosmanpasa Hospital, we retrospectively analyzed the charts of patients who were diagnosed with ureteral stone between February 2008 – September 2008. We evaluated patients diagnosed with a single distal ureteral stone which was shown with CT (n:148). Patient charts were evaluated and patients with peptic ulcer, urinary tract infection in diabetics, spontaneous stone passage history, long term colic pain and antihypertensive drug usage were not included. Stones with a diameter of 10 mm or below identified with CT were included into the evaluation. No patient with alpha blocker treatment was included in the study. Patients with missing data were not included and finally 88 patients were included and were grouped as smokers or non-smokers.

CT was performed using a MDCT (somatom sensation cardiac 64, Siemens Medical Solutions). Non-contrast images were obtained in prone position. The spiral scan time was 10–15 seconds, rotation time 0,5 seconds, slice width 1,5 mm and pitch factor was 1,2. Images were obtained from the diaphragms to the symphysis pubis. Stone diameter was calculated using the benchmark software of the CT. The longest diameter was taken into consideration.

The policy of our department is to follow patients with ureteral stones with the previously mentioned criteria for 4 weeks. During the 4 week period patients are asked to come weekly for evaluations and during this time they are asked to void in a separate container. At the end of 4 weeks if spontaneous passage does not occur we suggest a definitive treatment option. All patients included in the study had a record of weekly evaluation during the 4 week period. Non-steroid antiinflamatory drugs (diclophenac sodium 75 mg) was used only during painful renal colic episodes. The weekly check-up charts were controlled for stone passage, pain and NSAID consumption. Stone passage was defined as radiological proof of no stone or patient record of proof of the stone.

The effect of the stone size and smoking status on spontaneous passage was analyzed using multivariate and univariate analysis.

## Results

Total number of patients included in the study was 88. Female to male ratio was 2/7 (female: 20, male: 68) and median age was 40 (range: 17–77). Smoking habits was present in 30 (34%) patients (mean 13.7 pack-per-year). Spontaneous passage of the stone was observed in 69 (78%) patients. Mean age of patients with smoking habits was 42 ± 12 and female to male ratio was 0.33. In the group of patients without smoking habits the mean age was 43 ± 13 and female to male ratio was 0.34. The mean diameter of stones was similar in both groups of patients (smoking +: 4.7 ± 2.3 mm vs smoking -:4.7 ± 2.2 mm) (p > 0.05).

Spontaneous passage of stones, located at the distal portion of the ureter, within 4 weeks of follow-up was observed in 78% of patients. Spontaneous passage rates decreased as the stone burden increased. A detailed explanation of the stone passage rates according to stone burden and smoking habits is shown in Table 1.

**Table 1:** Spontaneous passage rates of distal ureteral stones according to stone burden, location and smoking habits

	**Spontaneous passage number (total number)**	**Spontaneous passage percentage**	**p Value**
All patients	69 (88)	78%	
1- 4 mm	42 (44)	95%	<0.05
4,1-8 mm	23 (36)	63%	
8,1-10 mm	4 (8)	50%	
Patients with smoking habits	23 (30)	76%	
1- 4 mm	17 (17)	100%	<0.05
4,1-8 mm	6 (10)	60%	
8,1-10 mm	0 (3)	0%	
Patients without smoking habits	46 (58)	79%	
1- 4 mm	25 (27)	92%	<0.05
4,1-8 mm	17 (26)	65%	
8,1-10 mm	4 (5)	80%	

Spontaneous passage rates were 76% and 79% in patients with smoking habits and no smoking habits respectively. There was no statistical difference between the two groups of patients (p > 0.05). Further analysis was made according to stone diameter. None of the patients with smoking habits who had a stone diameter between 8 and 10 mm were able to pass their stones within 4 weeks of follow-up. On the other hand patients with no smoking habits had an 80% passage rate of stones between 8 and 10 mm. However due to the low number of patients (smokers n:3 vs non-smokers n:5) in this group the differences could not be proven statistically. Spontaneous stone passage rates of stones between 1-4 mm and 4-8 mm showed no difference when patients were compared according to smoking habits (p > 0.05). Smoking density did not differ in smokers who required an intervention (mean 14 pack-per-year) for their stone or who passed their stone without any intervention (mean 13.7 pack-per-year) (p > 0.05).

## Discussion

According to the data of the World Health Organization (WHO), the mean frequency of tobacco smoking in Turkey was 31,2% (49,4% males & 17,6% females) in 2003 [6]. With such high smoking rates it is obvious that smoking is a major health problem in Turkey which requires attention. However, this epidemiologic information is valuable in assessing its possible effects on various systems. In our study we evaluated the possible effects of smoking on spontaneous passage rates of distal ureteral stones. Previous animal studies results have shown that nicotine increases ureteral contraction frequency [5,7].

Ureteral stones are most often encountered in the distal portion. The treatment options for stones at this location differ between interventional management and watchful waiting according to the stone dimension. For stones left for spontaneous passage medical expulsive therapy option should be kept in mind. When initiating such a treatment the consequences of obstruction on kidney functions should be known and the period of watchful waiting should be limited with 4 weeks [1].

In the guideline published by EAU in 2013, spontaneous passage rates of stones was 68% and 95% consecutively for stones below 5 mm and 4 mm independent of location [1]. However the same guideline states that these rates significantly differ according to the stone location within the ureter. Spontaneous passage rates of stones below 4 mm in the proximal, mid and distal portions of the ureter were 25%, 45% and 70% respectively. A 5 mm cut-off value was used in the joint guideline of EAU and American Urological Association (AUA) published in 2007 [8]. According to these guidelines spontaneous passage rates were 68% for stones smaller than 5 mm and interventional treatment is advised in stones greater than 5 mm independent of the location the within the ureter. However location specific spontaneous passage rates are not stated in this guideline. Location of the stone within the ureter has been evaluated by some studies and all confirm that stone passage rates are higher in the distal portion [9,10]. However, detailed evaluations and solid date with respect to stone site and stone size evaluations are scarce.

The subject of medical expulsive treatment has been analyzed in a recently published meta analysis [11]. According to this meta analysis the additional benefit of medical expulsive treatment decreased as stone diameter decreased. The fact that spontaneous expulsion rates increased as stone diameter decreased was the reason why medical expulsive treatment did not have a significant effect in smaller stones. Alpha-blocker studies have shown that stone sizes ≥5 mm demonstrated a significant benefit from medical expulsive treatment [11]. In our study the spontaneous expulsion rates of stones ≥4 mm were lower in patients with smoking habits. The rate of spontaneous expulsion was 46% in smokers and 67% in non-smokers with stones ≥4 mm. However the spontaneous expulsion rate of stones <4 mm was similar in both groups. The data from our study implies that smoking may decrease spontaneous expulsion rates of stones larger than 4 mm.

Anti-edema drugs, spasmolitics, alpha-blockers, calcium channel blockers, prostaglandin inhibitors, glycerin trinitrate and steroids have been researched as an option for medical expulsive therapy [11]. The role of the adrenergic system has been emphasized in many of these studies. The main adrenergic agonist noradrenalin has a dose dependent relationship. Noradrenalin increases the frequency of peristalsis with it s positive chronotropic effect and creates ureteral obstruction with its inotropic effect that causes contraction in the smooth muscle cells of the ureter. Due to these reasons alpha adrenergic stimulus decreases the amount of urine passed through the ureter [12]. Although, the role of the adrenergic system and ureter have extensively been evaluated similar studies about the ach receptor activity is far more less. Especially as nicotine has an effect on the ureteral ach receptors similar studies about its effect may also be carried out.

Boyarsky et al. have shown that nicotine increases the peristaltic activity of the ureter in dogs. In their study they administered nicotine by intravenous, inhaled or topical route and shown that both intravenous and inhaled nicotine, although effective at different dosages, do alter the peristalsis of the ureter [5]. Nicotine most probably exhibits this effect on the ureter over the cholinergic receptors proven to be present in the ureter also [13]. Further studies have shown that in each breath of a cigarette smoked there is 120 μg nicotine and about 50% of this is absorbed to the circulation in humans. After a single breath of a cigarette the concentration of nicotine reaches 0,15-0,25 μg/ml in the arterial blood [14]. Subsequently a person consuming one cigarette within 1 hour will have 18,3 ng/ml of nicotine in their blood. The study of Boyarsky has stroked our attention and lately the increasing attention to medical expulsive treatments has led us to evaluate the effect of smoking on spontaneous expulsion of ureteral stones.

Our study has shown that spontaneous expulsion of stones is not affected from smoking in patients with stones <4 mm. However in the group of patients with stone diameter ≥4 mm the rate of spontaneous expulsion was lower in patients with smoking habits. These results should be further confirmed with studies including larger numbers of patients.

## Conclusion

Due to the small number of subjects neither a favorable nor un-favorable effect of smoking on spontaneous passage of distal ureteral stones has been shown. However, spontaneous passage rates in patients with a stone diameter of ≥4 mm was lower in smokers. These results should be further confirmed with studies including larger numbers of patients.

## Competing interests

The authors declare that they have no competing interests.

## Authors’ contribution

AF carried out the design of the study, drafting of the manuscript and interpretation of the results. YS carried out data collection, contributed to interpretation of the results and preparation of the manuscript. ZT carried out the statistical analysis, interpretation of the results and contributed to drafting of the manuscript. FOK contributed to critical revision of the manuscript for important intellectual content. SB contributed to critical revision of the manuscript for important intellectual content. MC contributed to critical revision of the manuscript for important intellectual content. All authors read and approved the final manuscript.

## Pre-publication history

The pre-publication history for this paper can be accessed here:

http://www.biomedcentral.com/1471-2490/14/27/prepub
